# Identification of PANoptosis-related signature reveals immune infiltration characteristics and immunotherapy responses for renal cell carcinoma

**DOI:** 10.1186/s12885-024-12067-2

**Published:** 2024-03-04

**Authors:** Yan Xu, Jingqi Hua, Hongliang Que, Tengyue Zeng, Quan Li, Junpeng Deng, Jianjun Xie

**Affiliations:** 1 Department of Urology, The First Hospital of China Medical University, Shenyang, 110001 China; 2 Department of Urology, Suzhou Municipal Hospital, Suzhou, 215001 China

**Keywords:** PANoptosis, Clear cell renal cell carcinoma, Papillary renal cell carcinoma, Chromophobe renal cell carcinoma, Tumor microenvironment, Immunotherapy

## Abstract

PANoptosis is a specific type of inflammatory programmed cell death (PCD) modality that can be involved in three key modes of cellular programmed cell death-pyroptosis, apoptosis and necroptosis. We analyzed PANoptosis activity in three common renal cell carcinoma subtypes (Clear cell renal cell carcinoma, Papillary renal cell carcinoma, and Chromophobe renal cell carcinoma) separately and constructed a new PANoptosis immunity index (PANII). In three renal cell carcinomas, we found that PANII was an effective predictor of immunotherapy efficacy in KIRC, KIRP and KICH, and the high PANII group was characterized by high immune infiltration and sensitivity to immunotherapy, while the low PANII group was prone to immune escape and immunotherapy resistance. We performed molecular docking prediction of each core protein comprising PANII and identified natural small molecule compounds with the highest affinity to target proteins. In addition, we found that down-regulation of PYCARD inhibited the proliferation and migration of renal clear cell carcinoma cells by in vitro functional assays, suggesting that PYCARD could be a novel target for renal clear cell carcinoma therapy. Our findings that the PANoptosis characterization-based index (PANII) helps to elucidate the tumor microenvironmental features of three common renal cell carcinoma subtypes and identify patient populations that will benefit from immunotherapy, providing a new tool for the clinical diagnosis and treatment of patients with intermediate- and advanced-stage renal cell carcinoma.

**Supplementary Information:**

The online version contains supplementary material available at 10.1186/s12885-024-12067-2.

## Background

Renal cell carcinoma is a common urological malignancy, which is composed of three main pathological subtypes, namely clear cell renal cell carcinoma (ccRCC, KIRC), papillary renal cell carcinoma (pRCC, KIRP), and renal chromophobe carcinoma (chRCC, KICH), with ccRCC being the most predominant subtype, accounting for approximately 75% of all pathologic types [1, 2]. Renal cell carcinoma has long been considered an immunogenic tumour resistant to conventional radiotherapy and chemotherapy, and its current main therapeutic strategy is surgery-based combination therapy and renal cell carcinoma is an immunotherapy-responsive tumour; immune checkpoint inhibition therapies targeting the immunosuppressive microenvironment have revolutionised cancer treatment; however, only a small proportion of patients derive lasting benefit from immune checkpoint inhibitors, which limits the use of these promising strategies in clinical practice [3–6]. Therefore, there is an urgent need to identify reliable molecular biomarkers to predict response to checkpoint blockade and improve the clinical efficacy of these therapies.

Apoptosis, a classical process of programmed tumor cell death, plays an important role in cancer suppression [7]. However, with the increase of resistance to chemotherapeutic drugs inducing apoptosis in tumor cells, more mechanisms of programmed cell death have been discovered, including Pyroptosis, Necrosis, Ferroptosis, Cuproptosis, Autophagy, and so on. Recently, a newly discovered programmed cell death pathway, in which the mechanisms of Pyroptosis, Apoptosis and Necroptosis are cross-linked, was named “PANoptosis”. Furthermore, PANoptosis cannot be characterized by any of the cell death modes of Pyroptosis, Apoptosis and Necroptosis alone ( [8–10]. PANoptosis in specific tumor types with the value of parsing tumour heterogeneity, but lack of PANoptosis on study of relationship between renal cell carcinoma.

In our study, PANoptosis was characterized by bioinformatics analysis for three common subtypes of renal cell carcinoma (KIRC, KIRP, and KICH) and a new metric, the PANoptosis Immunity Index (PANII), was constructed to assess the potential correlation between PANoptosis and the immune microenvironment of the three renal cell carcinoma subtypes and its predictive value for immunotherapy response. Our findings may provide innovative targeted therapies for the treatment of patients with renal cell carcinoma.

## Materials and methods

### Obtaining patient data on three renal cell carcinoma subtypes and identifying PANoptosis-related genes

We downloaded expression profiling data, clinical information, and pathology sections for KIRC, KIRP, and KICH patients from The Cancer Genome Atlas (TCGA) (https://portal.gdc.cancer.gov/) database (Deletion of sample data with incomplete survival data) [11]. Single-cell datasets GSE154763, GSE159913, GSE111360, GSE121636, GSE139555, GSE159115, GSE171306 and GSE159115 were downloaded from the GEO database and normalised. from the GSEA gene set, KEGG, Hallmark, and review articles. key regulatory genes for apoptosis, pyroptosis and necroptosis as PANoptosis-related genes, the final gene list was the tandem regulatory genes for apoptosis, pyroptosis and necroptosis [12, 13] (Table S1).

### Unsupervised clustering of PANoptosis-related genes

We used the R package “ConsensusClusterPlus” to implement consensus clustering based on PANoptosis-related genes to identify KIRC, KIRP and KICH subtypes [14]. The parameter settings were “maxK” set to “10”, “clusterAlg” set to “km “, “clusterAlg” is set to “km”, and “distance” is set to “pearson“ [15–17].

### Gene set enrichment analysis (GSEA)

We obtained reference genomes (Hallmark, c5go and c2kegg) from the Molecular Signature Database (MSigDB). The R package “clusterProfiler” was used to identify Hallmark, c5go and c2kegg biological pathways [18]. Screening conditions were |NES| > 1, NOM *p*-value < 0.05.

### Construction of the PANoptosis immunity index (PANII)

After cross-linking the key regulatory genes for apoptosis, pyroptosis and necroptosis, we retained the genes identified as “confirmed” by using the Boruta algorithm. Principal component analysis (PCA) was used to reduce the dimensionality of the resulting PANoptosis gene clusters. Subsequently, a PANoptosis Immunity Index (PANII) score was assigned to each patient by calculating the score for each sample using the following formula: Score = ∑PCA A - ∑PCA B [19]. Taking the median value, each patient was categorized into a high PANII group and a low PANII group.

### Analysis of the immune microenvironment

Tumor purity, ESTIMATE score, immune cell score, and stroma score were calculated for each sample using the R package " ESTIMATE " [20]. The single sample gene set enrichment analysis (ssGSEA) algorithm was used to study the level of immune infiltration based on different immune cell types. Lymphocyte scores in pathology sections were graded using a semi-quantitative scoring system (0–5) to describe tumor inflammation.

### Immunotherapeutic response

The Tumor Immune Dysfunction and Exclusion (TIDE) algorithm can be used to infer patient response to immunotherapy [21]. In addition we downloaded anti-PD-1 and anti-CTLA4 IPS scoring data from ccRCC via the TCIA database (https://tcia.at/home) [22] to assess patient response to immune checkpoint inhibitors.

### Molecular docking

Schrödinger software was used to screen small molecule compounds with high affinity to target proteins. Protein structures of target proteins (BAX-6EB6, CASP1-5MTK, CASP8-4PS1, and PYCARD-5H8O) were downloaded from the PDB database. Natural small molecule drugs were collected from the PubChem database (https://pubchem.ncbi.nlm.nih.gov/). We set Use PROPKA pH to 7.0 and energy minimization of the protein structure, docking using OPLS-2005 force field, Precision to standard precision, and simulated the binding poses of BAX, CASP1, CASP8, and PYCARD with the small molecule drugs by the Glide module in Schrödinger software.

### Immunohistological chemical staining

Human Protein Atlas Database (https://www.proteinatlas.org/) [23] to obtain histological validation of BAX, CASP1, CASP8 and PYCARD at the protein level between renal clear cell carcinoma tissues and normal kidney tissues.

### Cell culture

The human renal clear cell carcinoma cell lines 769-P and 786-O were purchased from the Shanghai Cell Bank of the Chinese Academy of Sciences and used. The cells were both cultured in medium containing 5% fetal bovine serum and at 37 °C with 5% carbon dioxide.

### Cell counting kit-8 (CCK8) cell activity assay and plate cloning assay

CCK8 and plate cloning were used to determine cell proliferative capacity. Cells were digested and resuspended into cell suspension and added to 96-well plates, CCK8 solution was added and incubation was continued for 4 h until a distinct orange color appeared, and absorbance at 450 nm was measured using an enzyme marker. Monolayer cultured cells in logarithmic growth phase were taken and blown into individual cell suspension by trypsin digestion and then counted. The cell suspensions were inoculated in Petri dishes at the appropriate cell density, followed by incubation at 37 °C with 5% CO2 for 2 weeks. Pure methanol was added for fixation. The fixative was removed, stained with Giemsa’s staining solution, washed with running water to remove the staining solution, air-dried, and photographed and counted using a fluorescence microscope.

### Transwell and wound-healing experiments

Transwell and Wound-healing assays were performed to determine cell invasive capacity. Cells were starved for 24 h and then digested and centrifuged to make cell suspension. Culture medium was added to the lower chamber of the 24-well plate, and the cell suspension was taken and added to the upper chamber and put into the incubator for 24 h for fixation and staining, after which the cells were observed and counted. Cells were inoculated in 6-well plates, scribed with a lance tip, and put into the incubator for 48 h for taking pictures.

### Statistical analysis

Survival curves were plotted using the Kaplan-Meier method to compare the difference in survival between the two groups. Receiver Operation Characteristic (ROC) curves, and univariate and multivariate Cox analyses were used to assess the prognostic value of the characteristics. Spearman correlation analysis was used to assess correlation. *p*-value ≤ 0.05 was considered statistically significant. All statistical analyses were performed by R.

## Results

### Identifying PANoptosis patterns in three renal cell carcinoma subtypes

**Fig. 1:**
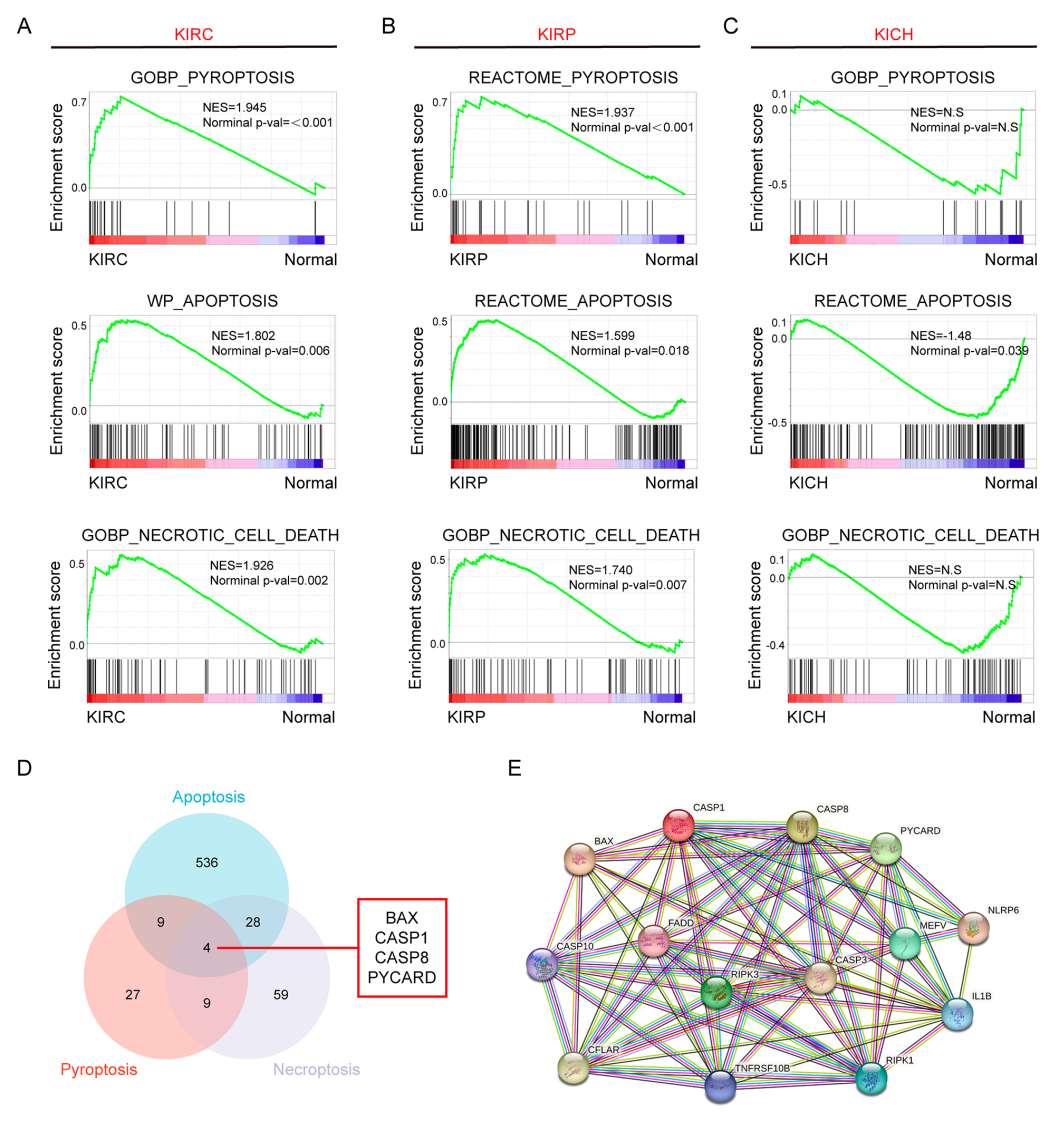
PANoptosis pattern analysis of three renal cell carcinoma subtypes. KIRC (**A**), KIRP (**B**) and KICH (**C**) Tumor tissues and normal renal tissues were enriched in Pyroptosis, Apoptosis and Necrotic cell death pathway enrichment results. (**D**) Crosstalk of key regulatory genes for Apoptosis, Pyroptosis and Necroptosis. (**E**) PPI network of PANoptosis core-associated genes

To investigate the PANoptosis patterns of three common renal cell carcinoma subtypes (KIRC, KIRP and KICH), we first performed functional enrichment analysis on tumor and normal kidney tissues, respectively. It was found that for KIRC and KIRP, tumor cells were significantly enriched in Pyroptosis, Apoptosis and Necrotic cell death pathways compared to normal tissues (*p* < 0.05). However, for KICH, tumor tissues were significantly enriched in the Apoptosis pathway (*p* < 0.05), while not significantly enriched in the Pyroptosis and Necrotic cell death pathways, it is therefore inferred that the PANoptosis process as well as the immune microenvironment is more active in KIRC and KIRP tumours. (Fig. 1A-C). We subsequently collected key regulatory genes for apoptosis, pyroptosis and necroptosis through GSEA gene set, KEGG, Hallmark and review articles and performed tandem linkage to serve as PANoptosis-related genes, including BAX, CASP1, CASP8 and PYCARD (Fig. 1D). Mapping the protein-protein interaction (PPI) network of the core PANoptosis-associated genes through the STRING website showed that BAX, CASP1, CASP8 and PYCARD were at the core of the whole network (Fig. 1E).

### PANoptosis clustering analysis in three renal cell carcinoma subtypes

**Fig. 2:**
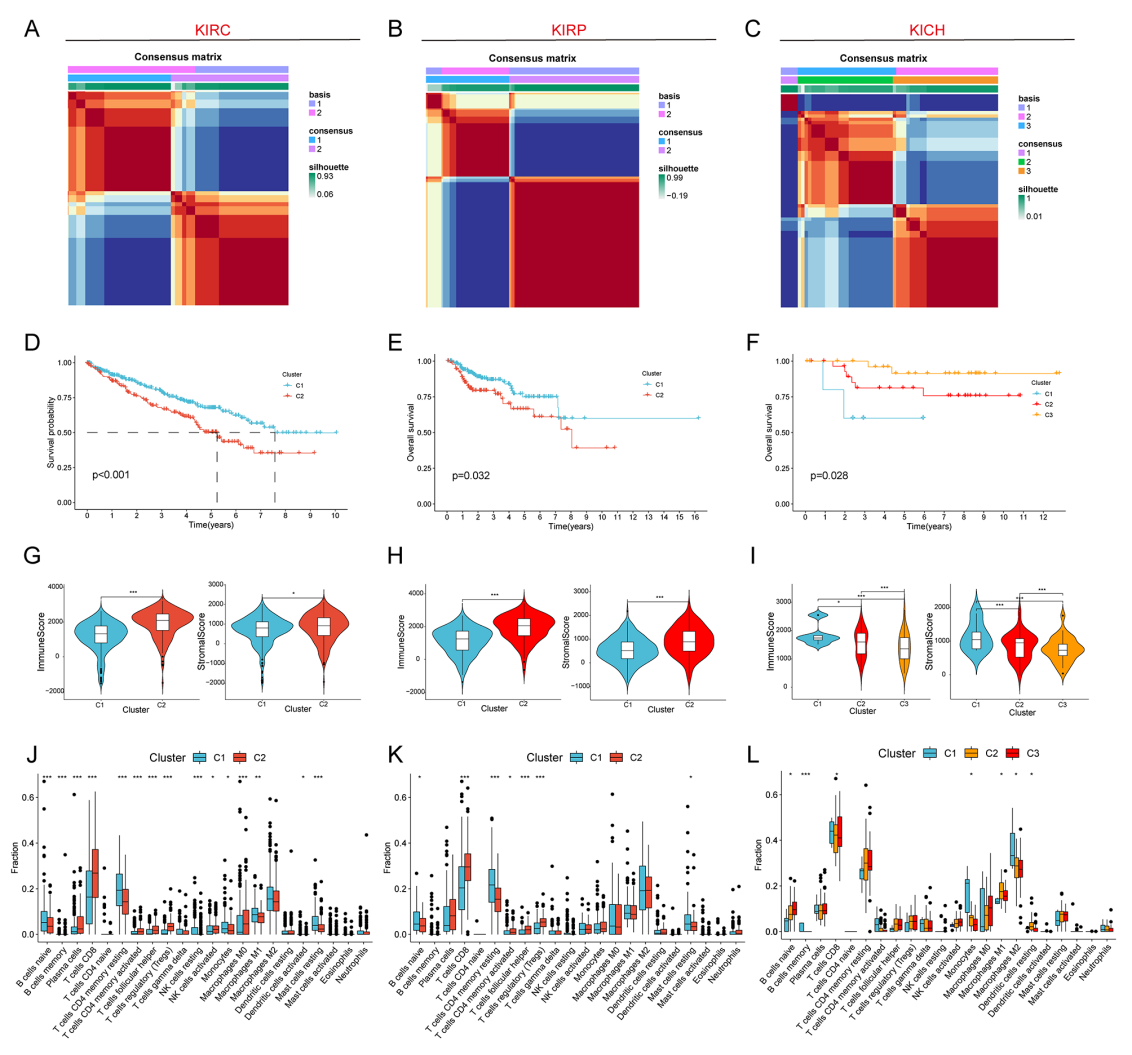
Cluster analysis of PANoptosis patterns. (**A**-**C**) Unsupervised cluster analysis of KIRC, KIRP and KICH. (**D**-**F**) Kaplan-Meier survival curves between different Clusters in KIRC, KIRP and KICH. (**G**-**I**) Differences in immunity scores and stromal scores between Clusters in KIRC, KIRP and KICH. (**J**-**L**) Differences in infiltration abundance of immune cells between different Clusters in KIRC, KIRP and KICH. Note * *p* < 0.05, **p < 0.01, ***p < 0.001

Based on the PANoptosis genes obtained from the above analyses, we performed consensus clustering of KIRC, KIRP and KICH, respectively, and classified KIRC and KIRP into 2 Clusters and KICH into 3 Clusters (Fig. 2A-C). Kaplam-Meier survival analyses showed significant differences in survival rates among Clusters in the three renal cell carcinoma subtypes. There was a significant difference in survival. survival was worst in Cluster 2 in KIRC and KIRP, while Cluster 1 patients in KICH had the worst survival (Fig. 2D-F). We then analyzed the differences in the immune microenvironment between the Clusters, and the results showed that Cluster 2 in KIRC and KIRP had significantly higher immune scores and stromal scores than Cluster 1, whereas Cluster 1 in KICH had significantly higher immune scores and stromal scores than the other two Clusters (Fig. 2G-I). The infiltration abundance of immune cells was assessed by the CIBERSORT algorithm, and CD8 T cells were significantly different in the three renal cell carcinoma Clusters, with higher levels of immune infiltration for Cluster 2 than for Cluster 1 in KIRC and KIRP, and the highest level of immune infiltration for Cluster 1 in KICH (Fig. 2J-L).

### Single-cell sequencing data analysis

To further investigate how PANoptosis-related genes are expressed in various cell types in the immune microenvironment of renal cell carcinoma, we analyzed single-cell sequencing data from mixed renal cell carcinoma (KIPAN), KIRC and KICH. Firstly, the dimensionality of the single-cell dataset is reduced by the UMAP method thereby illustrating the distribution of the single-cell sequencing profiles, where the size of the dots in the picture indicates the expression of the genes in the cell, and the color shades indicate the level of expression of the genes. In KIRC, BAX was predominantly expressed in malignant tumor cells, CD8 T cells, NK cells, and monocytes, CASP1 was predominantly expressed in monocytes and macrophages, CASP8 was highly expressed predominantly in CD8 T cells and NK cells, and PYCARD was predominantly expressed in monocytes and macrophages (Figure S1A-E). In KICH, BAX was predominantly expressed in Malignant, Endothelial, Pericytes, and monocyte macrophages, and PYCARD was predominantly expressed in monocyte macrophages (Figure S1F) In addition, we also plotted a heatmap of the expression of BAX, CASP1, CASP8, and PYCARD in the single-cell dataset (Figure S2). These results indicate that BAX, CASP1, CASP8 and PYCARD, which construct PANII, are all produced by various immune cells expressed in the tumour immune microenvironment and play a role in the renal cell carcinoma tumour microenvironment.

### Construction of the PANoptosis immunity index (PANII) and immune characterization of different renal cell carcinoma subtypes

**Fig. 3:**
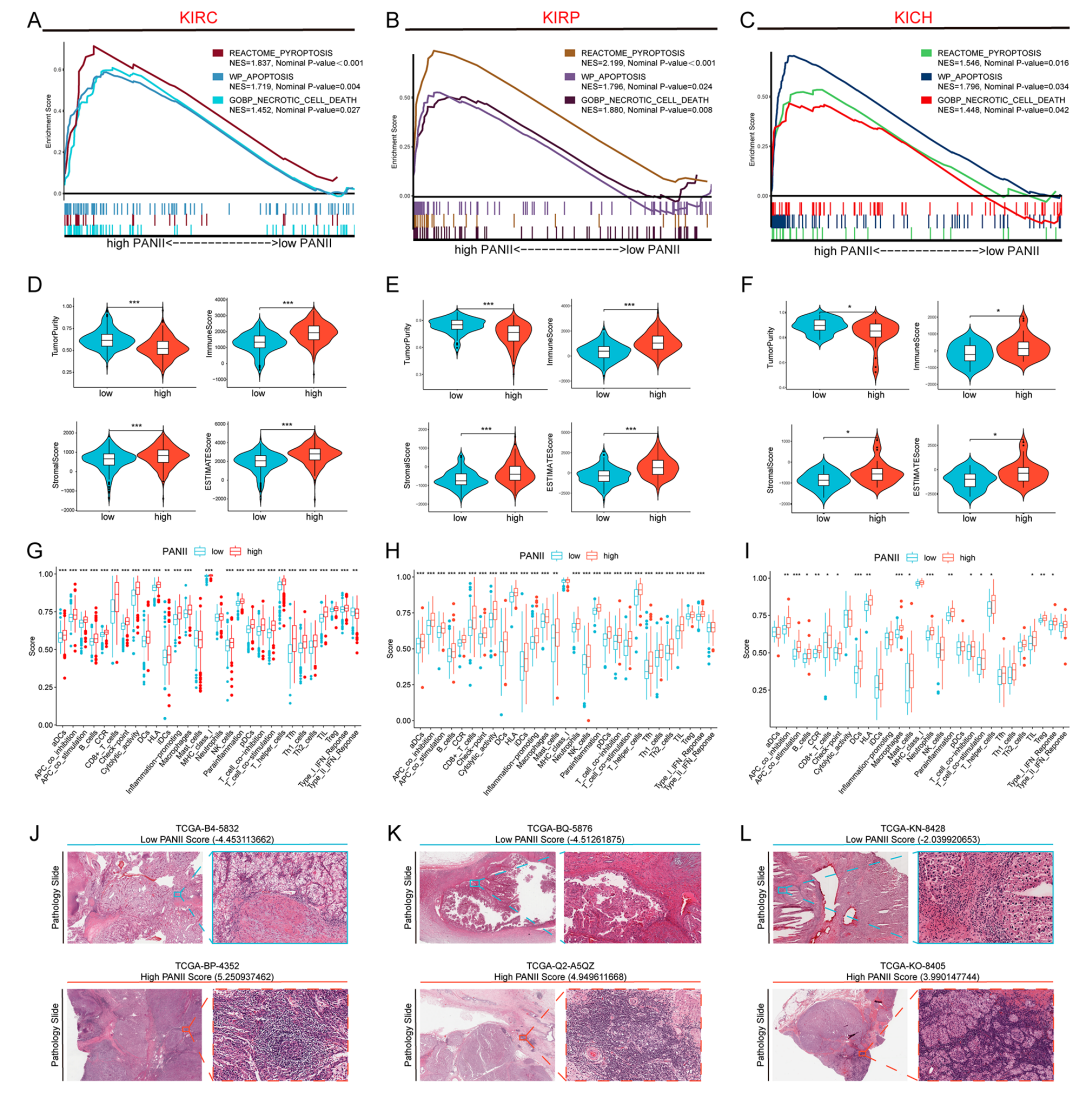
Enrichment analysis and immunological characterization of different PANII groups. GSEA analysis of high PANII groups in KIRC (**A**), KIRP (**B**) and KICH (**C**). Differences in TumorPurity, ImmuneScore, StromalScore and ESTIMATEScore between high/low PANII groups in KIRC (**D**), KIRP (**E**) and KICH (**F**). Differences in immune cells and immune function between patients in the high/low PANII groups in KIRC (**G**), KIRP (**H**), and KICH (**I**). Pathologic sections showing the level of immune cell infiltration in the high/low PANII groups in KIRC (**J**), KIRP (**K**) and KICH (**L**). Note * *p* < 0.05, **p < 0.01, ***p < 0.001

To further characterize the immune microenvironment of three common renal cell carcinoma subtypes (KIRC, KIRP and KICH) based on PANoptosis, we constructed PANoptosis by principal component analysis (PCA) based on the PANoptosis genes obtained from the above analysis, including BAX, CASP1, CASP8, and PYCARD characterization and derived a new index, the PANoptosis immunity index (PANII). The results of enrichment analysis by GSEA showed that the high PANII group was significantly enriched in Pyroptosis, Apoptosis and Necrotic cell death pathways in KIRC, KIRP and KICH, indicating that the PANoptosis signaling pathway was significantly active in the high PANII group compared to the low PANII group (Fig. 3A- C).

We analyzed the immune microenvironment characteristics of KIRC, KIRP, and KICH by PANII. Compared with the low PANII group, the high PANII group had lower TumorPurity, while ImmuneScore, StromalScore and ESTIMATEScore were all increased (Fig. 3D-F). We also analyzed the immune characteristics of different PANII groups by immune cells and immune functions, and the results showed that most of the immune cells and immune functions were significantly higher in the high PANII group than in the low PANII group (Fig. 3G-I). In addition, we further confirmed the above immune cell infiltration characteristics by pathological sections. In KIRC, the level of immune cell infiltration was higher in the high PANII group (TCGA-BP-4352) than in the low PANII group (TCGA-B4-5832) (Fig. 3J). In KIRP, the level of immune cell infiltration was higher in the high PANII group (TCGA-Q2-A5QZ) than in the low PANII group (TCGA-BQ-5876) (Fig. 3K). In KICH, the level of immune cell infiltration was higher in the high PANII group (TCGA-KO-8405) than in the low PANII group (TCGA-KN-8428) (Fig. 3L).

### Association of PANII with the efficacy of immunotherapy for subtypes of renal cell carcinoma

**Fig. 4:**
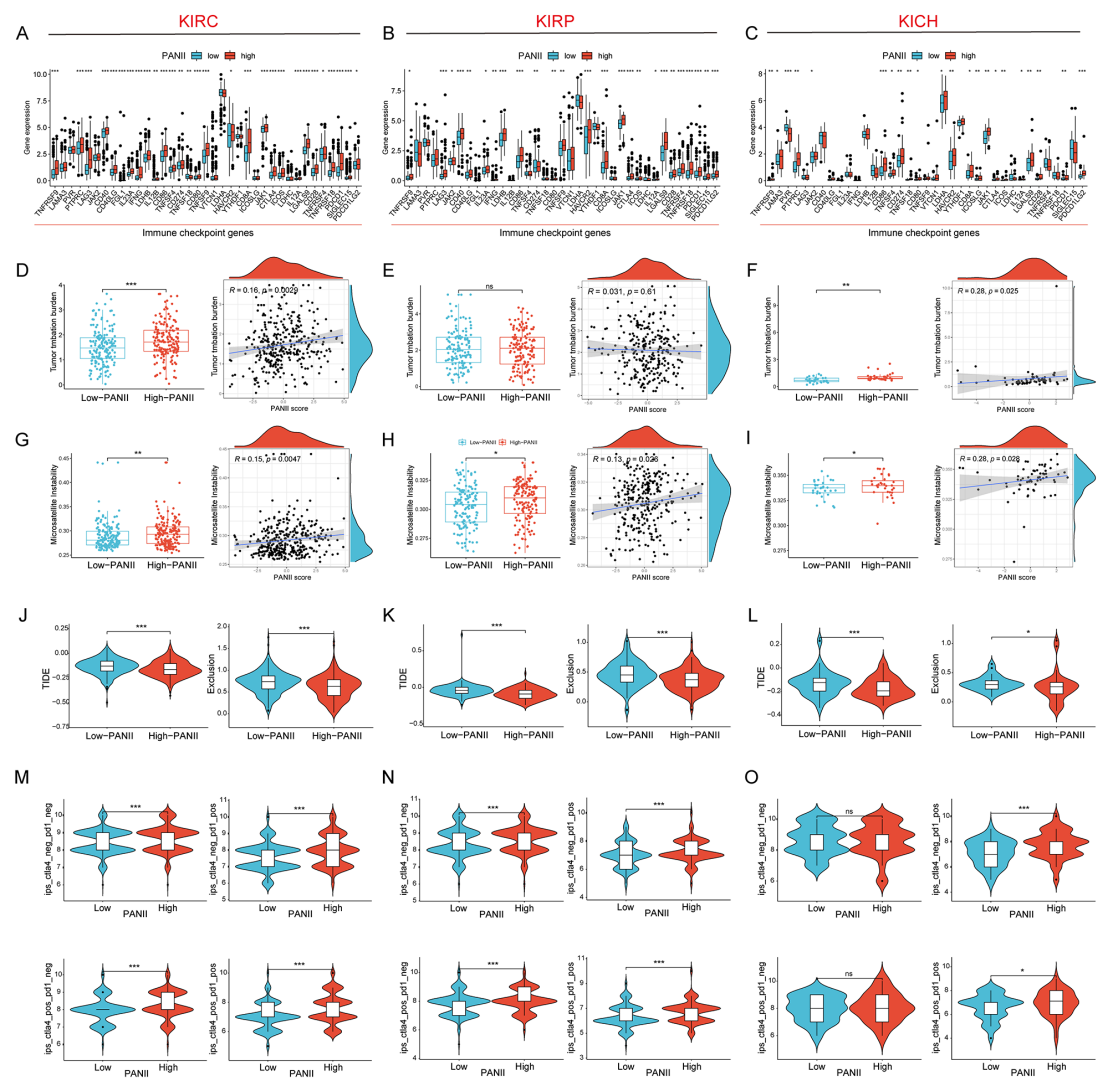
Association of PANII with immunotherapy outcome. (**A**-**C**) Differences in common immune checkpoint expression between patients in high/low PANII groups in KIRC, KIRP, and KICH. Differences in TMB levels (**D**-**F**) and MSI levels (**G**-**I**) between patients in high/low PANII groups in KIRC, KIRP, and KICH. (**J**-**L**) Differences in TIDE and Exclusion levels between patients in high/low PANII groups in KIRC, KIRP and KICH. (**M**-**O**) IPS scores for anti-PD1(-)CTLA4(-), anti-PD1(+)CTLA4(-), anti-PD1(-)CTLA4(+), and anti-PD1(+)CTLA4(+) blocker ground IPS scores for the high and low PANII groups in KIRC, KIRP, and KICH

We next analyzed the association between PANII and immune checkpoints, and found that in KIRC, KIRP and KICH, patients in the high PANII group significantly over-expressed common immune checkpoints, which, combined with the above analysis of the immune microenvironment, suggests that in the three renal cell carcinoma subtypes mentioned above, patients in the high PANII group exhibited a “hot “tumor microenvironment, i.e., they might be more sensitive to immunotherapy (Fig. 4A-C). Tumor mutational load (TMB) refers to the number of mutations in tumor cells; the higher the TMB, the more effective the immunotherapy [24]. Microsatellite instability (MSI) is a highly mutated phenotype, and MSI is associated with increased neoantigenic load in tumors, thus making them sensitive to ICI treatment [25]. By analyzing the association of TMB, MSI and PANII, we found that in KIRC and KICH, TMB and MSI showed a significant positive correlation with PANII and were significantly higher in patients in the high PANII group than in the low PANII group. However, in KIRP, PANII showed a significant positive correlation with MSI and no significant correlation with TMB (Fig. 4D-I). Patients with lower TIDE scores were more likely to benefit from immunotherapy [21], and through the correlation analysis of PANII with TIDE, we found that in KIRC, KIRP, and KICH, the high PANII group had significantly lower TIDE score and Exclusion were significantly lower than those of the low PMGI group, and it can be inferred that the high PMGI group responded better to immunotherapy (Fig. 4J-L). Immunogenicity was assessed by immunophenotypic core (IPS) scoring to predict patient response to immune checkpoint blockade (anti-PD1 and/or anti-CTLA4), with higher IPS scores indicating better predicted immunotherapy outcomes.

We found that in KIRC, KIRP, and KICH, patients in the high PANII group had significantly higher scores for anti-PD1/CTLA4 immunotherapy than those in the low PANII group (Fig. 4M-O). Subsequent validation of VMRI for predicting immunotherapy efficacy by external immunotherapy datasets showed that patients in the immunotherapy-responsive group in the Imvigor210 (anti-PD-L1) and Kim (anti-PD-1) cohorts had significantly higher PANII values than patients in the non-responsive group (Figure S3 A-B). All these results indicated that PANII could better predict the immunotherapy effect in three common renal cellcarcinoma subtypes (KIRC, KIRP, and KICH), and the high PANII group responded better to immunotherapy.

### Molecular docking of core target proteins

**Fig. 5:**
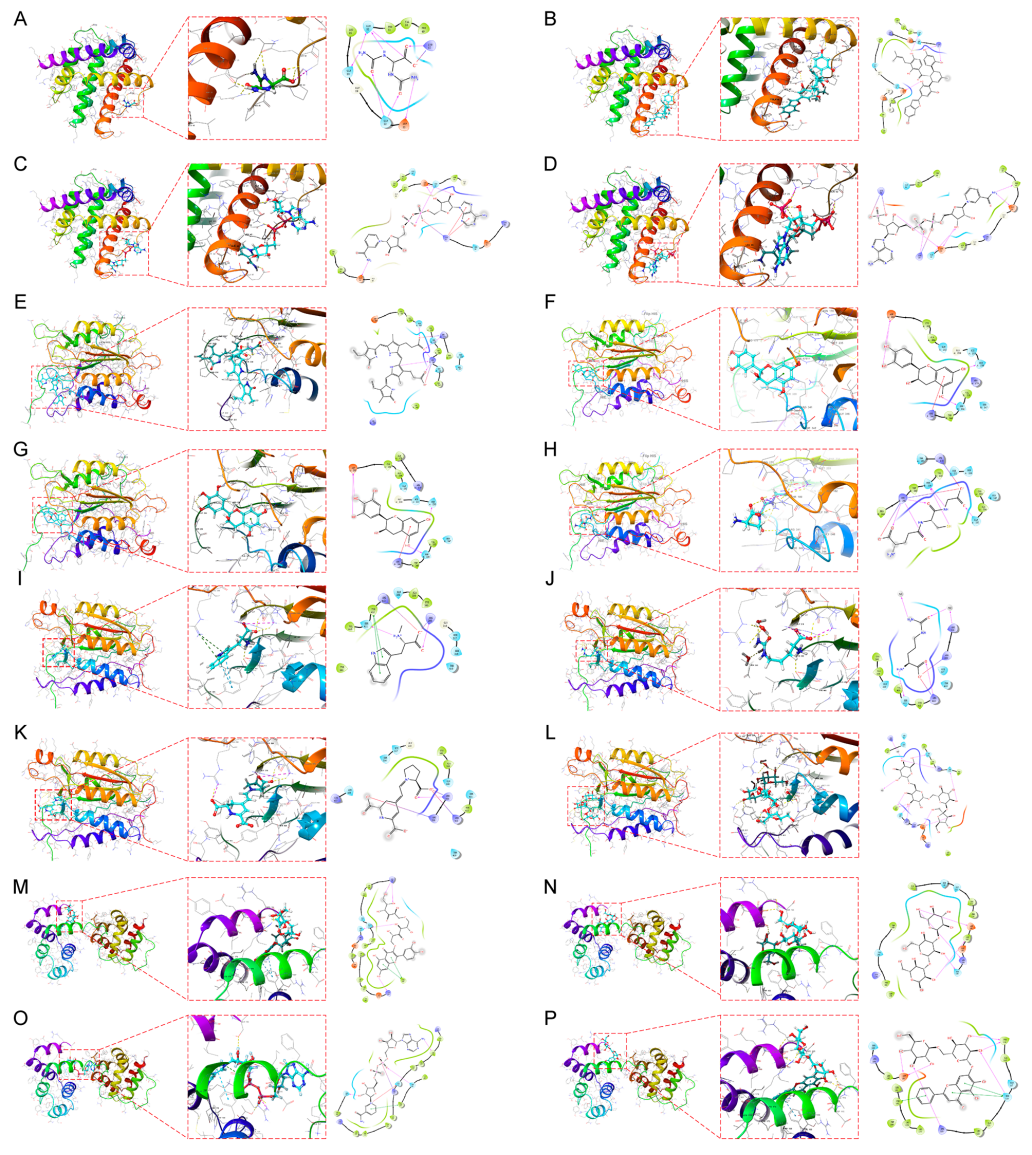
Molecular docking. Screening of candidate small molecules for target proteins using molecular docking. The figure shows the docking poses of the BAX active pocket with Allantoic Acid (**A**), Chalcomoracin (**B**), Nadide (**C**), and Triphosphopyridine Nucleotide (**D**). CASP1 active pocket with Biliverdin (**E**), Epicatechin ( **F**), Epigallocatechin (**G**), and Glutathione (**H**). docking poses of CASP8 active pocket with Abrine (**I**), Citrulline (**J**), Indicaxanthin (**K**), and Stachyose (**L**). docking poses of PYCARD active pocket with Heliosin (**M**), Laminaran (**N**), Nadide (**O**) and Oroxin B (**P**) in docking poses

Molecular docking is a computational algorithm for structure-based compound screening, which is a combination of core target and structure-based approach to find the feasibility of a drug candidate. We obtained protein structures of BAX (6EB6), CASP1 (5MTK), CASP8 (4PS1) and PYCARD (5H8O) from PDB database for molecular docking with natural small molecule compounds. The top four small molecules (Allantoic Acid, Chalcomoracin, Nadide, and Triphosphopyridine Nucleotide) with the highest binding affinity to the BAX binding pocket (Fig. 5A-D), the top four small molecules with the highest binding affinity to the CASP1 binding pocket (Biliverdin, Epicatechin, Epigallocatechin, and Glutathione) (Fig. 5E-H), the top four small molecules with the highest binding pocket binding to CASP8 (Abrine, Citrulline, Indicaxanthin, and Stachyose) (Fig. 5I-L), and the top four small molecules with the PYCARD top four small molecules (Heliosin, Laminaran, Nadide, and Oroxin B) with the highest binding pockets (Fig. 5M-P). For example, Allantoic Acid forms hydrogen bonds with BAX amino acid residues Gln-28, Gln-32, Asp-33, Gln-52, and Lys-57, with Gln-28, Asp-33, and Gln-52 acting as hydrogen bond acceptors and Gln-32 and Lys-57 acting as hydrogen bond donors.

### Immunohistological chemical staining

We first compared the differences in protein expression of BAX, CASP1, CASP8 and PYCARD in renal clear cells by CPTAC database, and the results showed that the protein levels of BAX, CASP1, CASP8 and PYCARD were significantly higher than those in normal tissues in tumor cells (Figure S4A). Later we also verified the above results by immunohistochemical staining results of BAX, CASP1, CASP8 and PYCARD in renal normal tissues and renal clear cell carcinoma (Figure S4B).

### Knockdown of PYCARD inhibits the proliferation and migration of renal clear cell carcinoma cells

**Fig. 6:**
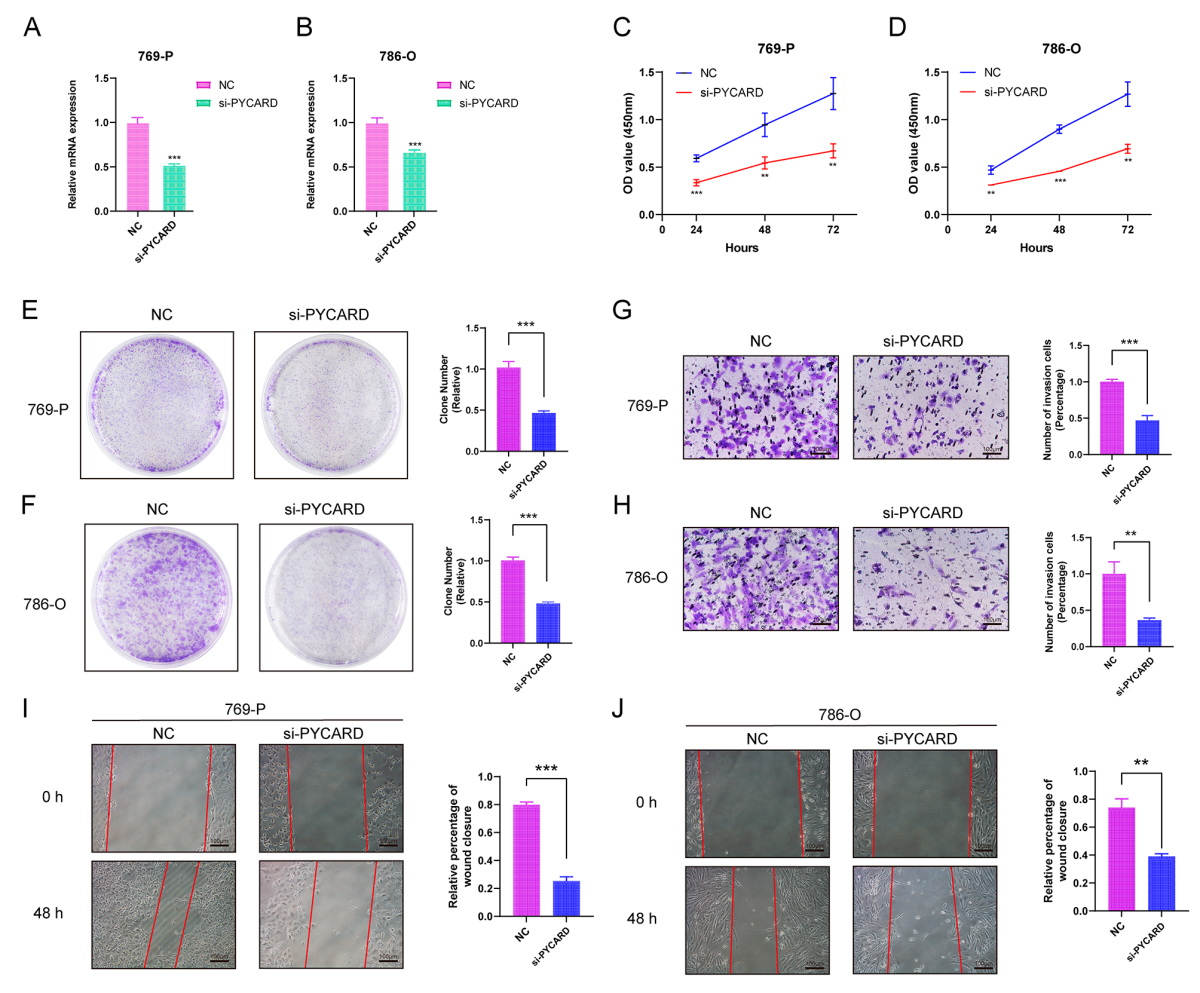
Knockdown of PYCARD inhibits renal clear cell carcinoma cell proliferation and migration. (**A**-**B**) QPCR verified the mRNA expression level of PYCARD in 769-P and 786-O cells after transfection with siRNA. CCK8 experiments(**C**-**D**), Plate cloning assay (**E**-**F**), Transwell cell migration ability (**G**-**H**) and Wound-healing assay (**I**-**J**) of normal renal tissues and 769-P and 786-O cells after transfection with si-PYCARD. Note * *p* < 0.05, **p < 0.01, ***p < 0.001

We designed siRNA for PYCARD to silence PYCARD expression in human renal clear cell carcinoma cell lines 769-P and 786-O cells to investigate the role of PYCARD in renal clear cell carcinoma. The silencing effect of PYCARD was detected by QPCR, and si-PYCARD could effectively knock down the expression of PYCARD (Fig. 6A-B). We further silenced PYCARD in human renal clear cell carcinoma cell lines 769-P and 786-O cells to investigate the role of PYCARD in renal clear cell carcinoma. The results of CCK8 and plate cloning experiments showed that the proliferative ability of 769-P and 786-O cells in the si-PYCARD group was significantly lower than that in the NC group (Fig. 6C-F). The results of Transwell assay showed that the cell migration ability of 769-P and 786-O cells was significantly reduced after knocking down PYCARD (Fig. 6G-H). Wound-healing assay showed that the migration ability of 769-P and 786-O cells in the si-PYCARD group was significantly lower than that of the NC group after 48 h (Fig. 6I-J). The above results indicated that the proliferation and migration of renal clear cell carcinoma cells were inhibited after knockdown of PYCARD.

## Discussion

An increasing number of studies have shown that cell death is an important anti-cancer defense mechanism and therapeutic target. A dynamic network of molecular interactions exists for tumor cells to escape the critical requirement for malignant cell survival and progression when cell death is evaded, in which PANoptosis is a complex mode of cell death with interconnections between cell deaths. Therefore, exploring the mechanisms andfunctions of cell death, especially the forms of PANoptosis and the regulatory mechanisms during cell death, will provide some insights for future cancer therapy [26–28]. In this study, we first analyzed three common renal cell carcinoma subtypes (KIRC, KIRP, and KICH) for the occurrence of PANoptosis in their tumor microenvironment and constructed a PANoptosis signature based on PANoptosis-related genes (BAX, CASP1, CASP8, and PYCARD) and derived a new metric, the The PANoptosis Immunity Index (PANII) can reflect the characteristics of PANoptosis in KIRC, KIRP and KICH, and among the three renal cell carcinoma subtypes mentioned above, the group with high PANII showed a “hot” tumor microenvironment, i.e., it was more effective for immunotherapy. Finally, we identified natural small molecules that can target PANoptosis-related target proteins by molecular docking and determined the role of PYCARD in renal clear cell carcinoma by in vitro functional assay.

The survival of tumor cells is closely related to the fact that the tumor microenvironment in which they reside helps them evade immune surveillance and drug interference [29]. We analyzed three common renal cell carcinoma subtypes (KIRC, KIRP, and KICH) and PANoptosis characteristics by ESTIMATE algorithm, ssGSEA algorithm, and pathological sections, respectively, and found that the high PANII group was highly correlated with immune cell infiltration and immune function. We then compared the differences in the expression levels of common immune checkpoints between the high and low PANII groups and showed that most were highly expressed in the PANII group. In addition, we also analyzed the association of TMB and MSI with PANII, suggesting that patients in the high PANII group with “hot” tumors in KIRC, KIRP, and KICH may be more effective for immunotherapy. Then we also showed that patients in the high PANII group were more effective for anti-PD-L1, anti-PD-1 and anti-CTLA-4 immunotherapy by immunotherapy response algorithms (TIDE and IPS). Finally, the results were further validated by immunotherapy datasets Imvigor210 (anti-PD-L1) and Kim cohort (anti-PD-1). The above results indicate that PANII can effectively evaluate the immunotherapy effects of three common renal cell carcinoma subtypes (KIRC, KIRP and KICH), which is important for the future precision treatment of renal cell carcinoma patients.

The PANII index was constructed by the incorporation of four PANoptosis genes, including BAX, CASP1, CASP8, and PYCARD. The proteins encoded by BAX belong to the BCL2 family of proteins, and members of the family play important roles as anti-apoptotic or pro-apoptotic factors involved in programmed cell death, and furthermore, it has been reported that the association between BAX and BCL2 is a key mechanism in determining the key mechanism for cell survival after apoptotic stimuli [30]. CASP1 and CASP8 encode proteins that are also members of the cysteine-aspartate protease (caspase) family, and sequential activation of caspases plays an important role in the execution phase of apoptosis. caspases exist as inactive zymogens on conserved protein hydrolytic processing on conserved aspartic acid residues, generating two subunits of size that dimerize to form the active enzyme, a process that has been shown to play an important role in the induction of apoptosis, especially Caspase-8 A key protein of cross-talk signal way in “PANoptosis " in cancer [8, 31–33]. PYCARD functions as a key mediator of apoptosis and inflammation and promotes cystatinase-mediated apoptosis, mainly involving cystatinase-8 and cystatinase-9, possibly in a cell type-specific manner [34, 35]. PYCARD is also involved in the transcriptional activation of cytokines and chemokines independent of inflammatory vesicles; this function may involve AP-1, NF-κB, MAPK and caspase-8 signaling pathways [36]. This study found that knockdown of PYCARD significantly inhibited the proliferation and invasion of renal clear cell carcinoma.

As another application of PANII efficacy prediction, we demonstrate the feasibility of a structure-based approach to find small molecule drug candidates that can target core proteins. Chalcomoracin, which has a strong affinity for BAX, has been shown to inhibit cell proliferation through endoplasmic reticulum stress-mediated paraptosis and to increase the sensitivity of non-small cell lung cancer to radiotherapy [37]. Of the top four small molecule drugs with the highest affinity for CASP1, Epicatechin has a significant role in the regulation of NADPH oxidase-dependent oxidant production and energy homeostasis [38]. Abrine has the highest affinity for the CASP8 docking pocket and has been shown to inhibit apoptosis of osteoblasts in osteoarthritis through the PIM2/VEGF signaling pathway. In addition, Abrine can target IDO1 to inhibit tumor cell immune escape and enhance anti-PD-1 immunotherapy in hepatocellular carcinoma [39, 40]. Laminaran has a high affinity for PYCARD and has been reported to act as a radiosensitizer and protective agent in melanoma [41].

Although our constructed PCDI can closely reflect the prognosis of renal clear cell carcinoma as well as predict drug sensitivity and treatment efficacy, certain limitations still exist in this study. First, the data for our analysis were obtained from public databases, which may have led to some case selection bias in case selection. Second, although we collected several external datasets to validate the conclusions obtained in this study, it is still necessary to collect a large amount of clinical case data for evaluation to further validate the accuracy of our findings. In addition, we only found natural small molecule drugs that can target BAX, CASP1, CASP8 and PYCARD through molecular docking, but no experimental validation was performed. Finally, further in vivo and in vitro experiments are needed to explore the specific mechanism and function of PANoptosis genes in renal cell carcinoma.

## Conclusion

In summary, through the comprehensive analysis of PANoptosis characteristics of three common renal cell carcinoma subtypes (KIRC, KIRP and KICH), we conclude that PANII can effectively reflect the immune microenvironmental status of KIRC, KIRP and KICH and predict the immunotherapeutic response of renal cell carcinoma patients. In addition, knockdown of PYCARD inhibited the progression of renal clear cell carcinoma cells, suggesting that PYCARD may be a potential target for the treatment of renal clear cell carcinoma. In an era when immunotherapy holds great promise for cancer treatment, PANII provides guidance for clinical diagnosis and individualized comprehensive treatment of renal cell carcinoma.

### Electronic supplementary material

Below is the link to the electronic supplementary material.

Supplementary Material 1

## Data Availability

All data utilized in this study are included in this article and all data supporting the findings of this study are available on reasonable request from the corresponding author.
