# Supplementary material for: Identification of PANoptosis-related signature reveals immune infiltration characteristics and immunotherapy responses for renal cell carcinoma
Source: BMC Cancer. 2024 Mar 4;24:292. doi: 10.1186/s12885-024-12067-2 (PMC10913266; doi:10.1186/s12885-024-12067-2)
Supplement: Supplementary file 1 — Supplementary Material 1 [file 12885_2024_12067_MOESM1_ESM.docx]

| Supplementary Table 1. The information of 672 genes related to PANoptosome | | | |
| --- | --- | --- | --- |
| **No.** | **Apoptosis** | **Pyroptosis** | **Necroptosis** |
| **1** | AATF | BAK1 | GLUD1 |
| **2** | ABL1 | BAX | GLUD2 |
| **3** | ACAA2 | CASP1 | ALOX15 |
| **4** | ACKR3 | CASP3 | FTH1 |
| **5** | ACVR1 | CASP4 | PYG |
| **6** | ACVR1B | CASP5 | CAPN1 |
| **7** | ADORA1 | CASP6 | CASP1 |
| **8** | AEN | CASP8 | GLNA |
| **9** | AGT | CASP9 | BAX |
| **10** | AGTR2 | CHMP2A | BCL2 |
| **11** | AIFM1 | CHMP2B | FADD |
| **12** | AKT1 | CHMP3 | RIPK1 |
| **13** | ANXA6 | CHMP4A | CHMP4C |
| **14** | APAF1 | CHMP4B | TNFRSF1A |
| **15** | APPL1 | CHMP4C | TRADD |
| **16** | AR | CHMP6 | TRAF2 |
| **17** | ARHGEF2 | CHMP7 | PPIA |
| **18** | ARL6IP5 | CYCS | CAPN2 |
| **19** | ARMC10 | ELANE | HSP90A |
| **20** | ARRB2 | GPX4 | FAS |
| **21** | ASAH2 | GSDMB | TNFSF6 |
| **22** | ATF3 | GSDMC | TNFRSF6 |
| **23** | ATF4 | GSDMD | CASP8 |
| **24** | ATM | GSDME | JNK |
| **25** | ATP2A1 | GZMB | JAK2 |
| **26** | ATP2A3 | HMGB1 | CAMK2 |
| **27** | ATP5IF1 | IL18 | FASLG |
| **28** | AVP | IL6 | IFNG |
| **29** | BAD | NOD2 | STAT3 |
| **30** | BAG3 | IRF1 | IRF9 |
| **31** | BAG5 | IRF2 | TNFSF10 |
| **32** | BAG6 | NLRC4 | TNFRSF10A |
| **33** | BAK1 | NLRP1 | TNFRSF10B |
| **34** | BAX | NLRP2 | CFLAR |
| **35** | BBC3 | NLRP3 | XIAP |
| **36** | BCAP31 | NLRP6 | BID |
| **37** | BCL10 | NLRP7 | AIFM1 |
| **38** | BCL2 | NOD1 | TRPM7 |
| **39** | BCL2A1 | PLCG1 | IFNAR1 |
| **40** | BCL2L1 | PJVK | IFNAR2 |
| **41** | BCL2L10 | PRKACA | IFNGR1 |
| **42** | BCL2L11 | PYCARD | IFNGR2 |
| **43** | BCL2L12 | SCAF11 | TLR3 |
| **44** | BCL2L14 | TINAP | TIRP |
| **45** | BCL2L2 | TIRAP | IFNA |
| **46** | BCL3 | TP53 | IFNB |
| **47** | BCLAF1 | TP63 | TRIF |
| **48** | BDKRB2 | AIM2 | VDAC1 |
| **49** | BDNF | GSDMA | SLC25A4S |
| **50** | BECN1 |  | PPID |
| **51** | BID |  | CYLD |
| **52** | BIK |  | RIPK3 |
| **53** | BIRC6 |  | MLKL |
| **54** | BLOC1S2 |  | TRAF5 |
| **55** | BMF |  | TLR4 |
| **56** | BMP4 |  | RBCK1 |
| **57** | BMP5 |  | HMGB1 |
| **58** | BMPR1B |  | JAK1 |
| **59** | BNIP3 |  | JAK3 |
| **60** | BNIP3L |  | TYK2 |
| **61** | BOK |  | STAT1 |
| **62** | BRCA1 |  | STAT2 |
| **63** | BRCA2 |  | STAT4 |
| **64** | BRSK2 |  | STAT5A |
| **65** | BTK |  | STAT5B |
| **66** | CAAP1 |  | STAT6 |
| **67** | CASP1 |  | H2A |
| **68** | CASP10 |  | TNFAIP3 |
| **69** | CASP12 |  | RNF31 |
| **70** | CASP2 |  | CHMP2A |
| **71** | CASP3 |  | CHMP2B |
| **72** | CASP4 |  | VPS24 |
| **73** | CASP5 |  | CHMP4A |
| **74** | CASP8 |  | CHMP4B |
| **75** | CASP8AP2 |  | CHMP6 |
| **76** | CASP9 |  | VPS4 |
| **77** | CAV1 |  | CHMP1 |
| **78** | CCAR2 |  | CHMP5 |
| **79** | CCK |  | SMPD1 |
| **80** | CD14 |  | PYCARD |
| **81** | CD24 |  | NLRP3 |
| **82** | CD27 |  | ZBP1 |
| **83** | CD28 |  | IL33 |
| **84** | CD38 |  | FTL |
| **85** | CD3E |  | SQSTM1 |
| **86** | CD44 |  | VDAC2 |
| **87** | CD5 |  | VDAC3 |
| **88** | CD70 |  | CHMP7 |
| **89** | CD74 |  | PGAM5 |
| **90** | CDIP1 |  | BIRC2 |
| **91** | CDKN1A |  | BIRC3 |
| **92** | CDKN2D |  | EIF2AK2 |
| **93** | CEBPB |  | PLA2G4 |
| **94** | CFLAR |  | DNM1L |
| **95** | CHAC1 |  | SPATA2 |
| **96** | CHCHD10 |  | FAF1 |
| **97** | CHEK2 |  | SHARPIN |
| **98** | CIB1 |  | NOX2 |
| **99** | CIDEB |  | USP21 |
| **100** | CLU |  | PARP1 |
| **101** | COA8 |  |  |
| **102** | COL2A1 |  |  |
| **103** | CRADD |  |  |
| **104** | CREB3 |  |  |
| **105** | CREB3L1 |  |  |
| **106** | CRH |  |  |
| **107** | CRIP1 |  |  |
| **108** | CSF2 |  |  |
| **109** | CSNK2A1 |  |  |
| **110** | CSNK2A2 |  |  |
| **111** | CTH |  |  |
| **112** | CTNNA1 |  |  |
| **113** | CTSC |  |  |
| **114** | CTTN |  |  |
| **115** | CUL1 |  |  |
| **116** | CUL2 |  |  |
| **117** | CUL3 |  |  |
| **118** | CUL4A |  |  |
| **119** | CUL5 |  |  |
| **120** | CX3CL1 |  |  |
| **121** | CX3CR1 |  |  |
| **122** | CXCL12 |  |  |
| **123** | CYLD |  |  |
| **124** | CYP1B1 |  |  |
| **125** | DAB2IP |  |  |
| **126** | DAP |  |  |
| **127** | DAP3 |  |  |
| **128** | DAPK1 |  |  |
| **129** | DAPK2 |  |  |
| **130** | DAPK3 |  |  |
| **131** | DAPL1 |  |  |
| **132** | DAXX |  |  |
| **133** | DBH |  |  |
| **134** | DCC |  |  |
| **135** | DDIAS |  |  |
| **136** | DDIT3 |  |  |
| **137** | DDIT4 |  |  |
| **138** | DDX3X |  |  |
| **139** | DDX47 |  |  |
| **140** | DDX5 |  |  |
| **141** | DEDD |  |  |
| **142** | DEDD2 |  |  |
| **143** | DELE1 |  |  |
| **144** | DEPTOR |  |  |
| **145** | DIABLO |  |  |
| **146** | DIDO1 |  |  |
| **147** | DNAJA1 |  |  |
| **148** | DNAJC10 |  |  |
| **149** | DNM1L |  |  |
| **150** | DPF2 |  |  |
| **151** | DYRK2 |  |  |
| **152** | E2F1 |  |  |
| **153** | E2F2 |  |  |
| **154** | EDA2R |  |  |
| **155** | EIF2AK3 |  |  |
| **156** | ELL3 |  |  |
| **157** | ENO1 |  |  |
| **158** | EP300 |  |  |
| **159** | EPHA2 |  |  |
| **160** | EPO |  |  |
| **161** | ERBB3 |  |  |
| **162** | ERCC6 |  |  |
| **163** | ERN1 |  |  |
| **164** | ERN2 |  |  |
| **165** | ERO1A |  |  |
| **166** | ERP29 |  |  |
| **167** | EYA1 |  |  |
| **168** | EYA2 |  |  |
| **169** | EYA3 |  |  |
| **170** | EYA4 |  |  |
| **171** | FADD |  |  |
| **172** | FAF1 |  |  |
| **173** | FAIM |  |  |
| **174** | FAIM2 |  |  |
| **175** | FAM162A |  |  |
| **176** | FAS |  |  |
| **177** | FASLG |  |  |
| **178** | FASTK |  |  |
| **179** | FBH1 |  |  |
| **180** | FBXW7 |  |  |
| **181** | FEM1B |  |  |
| **182** | FGA |  |  |
| **183** | FGB |  |  |
| **184** | FGF10 |  |  |
| **185** | FGFR1 |  |  |
| **186** | FGFR3 |  |  |
| **187** | FGG |  |  |
| **188** | FHIT |  |  |
| **189** | FIGNL1 |  |  |
| **190** | FIS1 |  |  |
| **191** | FNIP2 |  |  |
| **192** | FXN |  |  |
| **193** | FYN |  |  |
| **194** | FZD9 |  |  |
| **195** | G0S2 |  |  |
| **196** | GABARAP |  |  |
| **197** | GATA1 |  |  |
| **198** | GATA4 |  |  |
| **199** | GCLM |  |  |
| **200** | GDNF |  |  |
| **201** | GFRAL |  |  |
| **202** | GGCT |  |  |
| **203** | GHITM |  |  |
| **204** | GNAI2 |  |  |
| **205** | GNAI3 |  |  |
| **206** | GPER1 |  |  |
| **207** | GPX1 |  |  |
| **208** | GRINA |  |  |
| **209** | GSDME |  |  |
| **210** | GSK3A |  |  |
| **211** | GSK3B |  |  |
| **212** | GSKIP |  |  |
| **213** | GSTP1 |  |  |
| **214** | GZMB |  |  |
| **215** | HDAC1 |  |  |
| **216** | HERPUD1 |  |  |
| **217** | HGF |  |  |
| **218** | HIC1 |  |  |
| **219** | HIF1A |  |  |
| **220** | HINT1 |  |  |
| **221** | HIP1 |  |  |
| **222** | HIP1R |  |  |
| **223** | HIPK1 |  |  |
| **224** | HIPK2 |  |  |
| **225** | HMGB2 |  |  |
| **226** | HMOX1 |  |  |
| **227** | HNRNPK |  |  |
| **228** | HRAS |  |  |
| **229** | HRK |  |  |
| **230** | HSPA1A |  |  |
| **231** | HSPA1B |  |  |
| **232** | HSPB1 |  |  |
| **233** | HTRA2 |  |  |
| **234** | HTT |  |  |
| **235** | HYAL2 |  |  |
| **236** | HYOU1 |  |  |
| **237** | ICAM1 |  |  |
| **238** | IFI16 |  |  |
| **239** | IFI27 |  |  |
| **240** | IFI27L1 |  |  |
| **241** | IFI27L2 |  |  |
| **242** | IFI6 |  |  |
| **243** | IFNB1 |  |  |
| **244** | IFNG |  |  |
| **245** | IGF1 |  |  |
| **246** | IKBKE |  |  |
| **247** | IL12A |  |  |
| **248** | IL19 |  |  |
| **249** | ZNF385B |  |  |
| **250** | ZNF622 |  |  |
| **251** | IL2 |  |  |
| **252** | IL20RA |  |  |
| **253** | IL33 |  |  |
| **254** | IL4 |  |  |
| **255** | IL6R |  |  |
| **256** | IL7 |  |  |
| **257** | INCA1 |  |  |
| **258** | ING2 |  |  |
| **259** | ING5 |  |  |
| **260** | INHBA |  |  |
| **261** | INHBB |  |  |
| **262** | INS |  |  |
| **263** | ITGA6 |  |  |
| **264** | ITGAM |  |  |
| **265** | ITGAV |  |  |
| **266** | ITM2C |  |  |
| **267** | ITPR1 |  |  |
| **268** | ITPRIP |  |  |
| **269** | IVNS1ABP |  |  |
| **270** | JAK2 |  |  |
| **271** | JMY |  |  |
| **272** | JUN |  |  |
| **273** | KDM1A |  |  |
| **274** | KITLG |  |  |
| **275** | KRT18 |  |  |
| **276** | KRT8 |  |  |
| **277** | LCK |  |  |
| **278** | LGALS12 |  |  |
| **279** | LGALS3 |  |  |
| **280** | LRRK2 |  |  |
| **281** | LTBR |  |  |
| **282** | LY96 |  |  |
| **283** | MADD |  |  |
| **284** | MAEL |  |  |
| **285** | MAGEA3 |  |  |
| **286** | MAP2K5 |  |  |
| **287** | MAP3K5 |  |  |
| **288** | MAPK7 |  |  |
| **289** | MAPK8 |  |  |
| **290** | MAPK8IP1 |  |  |
| **291** | MAPK8IP2 |  |  |
| **292** | MAPK9 |  |  |
| **293** | MARCHF7 |  |  |
| **294** | MAZ |  |  |
| **295** | MCL1 |  |  |
| **296** | MDM2 |  |  |
| **297** | MELK |  |  |
| **298** | MFF |  |  |
| **299** | MIF |  |  |
| **300** | MIR132 |  |  |
| **301** | MIR15A |  |  |
| **302** | MIR16-1 |  |  |
| **303** | MIR17 |  |  |
| **304** | MIR198 |  |  |
| **305** | MIR21 |  |  |
| **306** | MIR210 |  |  |
| **307** | MIR221 |  |  |
| **308** | MIR222 |  |  |
| **309** | MIR26B |  |  |
| **310** | MIR27B |  |  |
| **311** | MIR449A |  |  |
| **312** | MKNK2 |  |  |
| **313** | MLH1 |  |  |
| **314** | MLLT11 |  |  |
| **315** | MMP9 |  |  |
| **316** | MNT |  |  |
| **317** | MOAP1 |  |  |
| **318** | MPV17L |  |  |
| **319** | MSH2 |  |  |
| **320** | MSH6 |  |  |
| **321** | MSX1 |  |  |
| **322** | MUC1 |  |  |
| **323** | MUL1 |  |  |
| **324** | MYBBP1A |  |  |
| **325** | NACC2 |  |  |
| **326** | NANOS3 |  |  |
| **327** | NBN |  |  |
| **328** | NCK1 |  |  |
| **329** | NCK2 |  |  |
| **330** | NDUFA13 |  |  |
| **331** | NDUFS3 |  |  |
| **332** | NFATC4 |  |  |
| **333** | NFE2L2 |  |  |
| **334** | NGF |  |  |
| **335** | NGFR |  |  |
| **336** | NKX3-1 |  |  |
| **337** | NLE1 |  |  |
| **338** | NME5 |  |  |
| **339** | NMT1 |  |  |
| **340** | NOC2L |  |  |
| **341** | NOG |  |  |
| **342** | NOL3 |  |  |
| **343** | NONO |  |  |
| **344** | NOS3 |  |  |
| **345** | NOX1 |  |  |
| **346** | NR4A2 |  |  |
| **347** | NUPR1 |  |  |
| **348** | OPA1 |  |  |
| **349** | P2RX4 |  |  |
| **350** | P2RX7 |  |  |
| **351** | P4HB |  |  |
| **352** | PAK2 |  |  |
| **353** | PAK5 |  |  |
| **354** | PARK7 |  |  |
| **355** | PARP1 |  |  |
| **356** | PARP2 |  |  |
| **357** | PAWR |  |  |
| **358** | PCGF2 |  |  |
| **359** | PDCD10 |  |  |
| **360** | PDCD5 |  |  |
| **361** | PDCD6 |  |  |
| **362** | PDIA3 |  |  |
| **363** | PDK1 |  |  |
| **364** | PDK2 |  |  |
| **365** | PDPK1 |  |  |
| **366** | PDX1 |  |  |
| **367** | PEA15 |  |  |
| **368** | PELI3 |  |  |
| **369** | PERP |  |  |
| **370** | PF4 |  |  |
| **371** | PHIP |  |  |
| **372** | PHLDA3 |  |  |
| **373** | PIAS4 |  |  |
| **374** | PIDD1 |  |  |
| **375** | PIH1D1 |  |  |
| **376** | PIK3R1 |  |  |
| **377** | PINK1 |  |  |
| **378** | PLAGL2 |  |  |
| **379** | PLAUR |  |  |
| **380** | PLEKHF1 |  |  |
| **381** | PLSCR3 |  |  |
| **382** | PMAIP1 |  |  |
| **383** | PML |  |  |
| **384** | POLB |  |  |
| **385** | POU4F1 |  |  |
| **386** | POU4F2 |  |  |
| **387** | PPARD |  |  |
| **388** | PPIA |  |  |
| **389** | PPIF |  |  |
| **390** | PPM1F |  |  |
| **391** | PPP1CA |  |  |
| **392** | PPP1R13B |  |  |
| **393** | PPP1R15A |  |  |
| **394** | PPP2R1B |  |  |
| **395** | PPP3CC |  |  |
| **396** | PPP3R1 |  |  |
| **397** | PRDX2 |  |  |
| **398** | PRELID1 |  |  |
| **399** | PRKCA |  |  |
| **400** | PRKCD |  |  |
| **401** | PRKDC |  |  |
| **402** | PRKN |  |  |
| **403** | PRKRA |  |  |
| **404** | PRODH |  |  |
| **405** | PSEN1 |  |  |
| **406** | PSMD10 |  |  |
| **407** | PSME3 |  |  |
| **408** | PTEN |  |  |
| **409** | PTGIS |  |  |
| **410** | PTH |  |  |
| **411** | PTPMT1 |  |  |
| **412** | PTPN1 |  |  |
| **413** | PTPN2 |  |  |
| **414** | PTPRC |  |  |
| **415** | PTTG1IP |  |  |
| **416** | PYCARD |  |  |
| **417** | QARS1 |  |  |
| **418** | RACK1 |  |  |
| **419** | RAF1 |  |  |
| **420** | RB1 |  |  |
| **421** | RB1CC1 |  |  |
| **422** | RBCK1 |  |  |
| **423** | RELA |  |  |
| **424** | RET |  |  |
| **425** | RFFL |  |  |
| **426** | RHOT1 |  |  |
| **427** | RHOT2 |  |  |
| **428** | RIPK1 |  |  |
| **429** | RIPK3 |  |  |
| **430** | RNF183 |  |  |
| **431** | RNF186 |  |  |
| **432** | RNF34 |  |  |
| **433** | RNF41 |  |  |
| **434** | RPL11 |  |  |
| **435** | RPL26 |  |  |
| **436** | RPS27L |  |  |
| **437** | RPS3 |  |  |
| **438** | RPS6KB1 |  |  |
| **439** | RPS7 |  |  |
| **440** | RRP8 |  |  |
| **441** | RTKN2 |  |  |
| **442** | RTL10 |  |  |
| **443** | S100A8 |  |  |
| **444** | S100A9 |  |  |
| **445** | SCG2 |  |  |
| **446** | SCN2A |  |  |
| **447** | SCRT2 |  |  |
| **448** | SELENOK |  |  |
| **449** | SELENOS |  |  |
| **450** | SENP1 |  |  |
| **451** | SEPTIN4 |  |  |
| **452** | SERINC3 |  |  |
| **453** | SERPINE1 |  |  |
| **454** | SFN |  |  |
| **455** | SFPQ |  |  |
| **456** | SFRP1 |  |  |
| **457** | SFRP2 |  |  |
| **458** | SGMS1 |  |  |
| **459** | SGPL1 |  |  |
| **460** | SGPP1 |  |  |
| **461** | SH3RF1 |  |  |
| **462** | SHH |  |  |
| **463** | SHISA5 |  |  |
| **464** | SIAH1 |  |  |
| **465** | SIAH2 |  |  |
| **466** | SIRT1 |  |  |
| **467** | SIVA1 |  |  |
| **468** | SKIL |  |  |
| **469** | SLC25A5 |  |  |
| **470** | SLC35F6 |  |  |
| **471** | SLC9A3R1 |  |  |
| **472** | SMAD3 |  |  |
| **473** | SNAI1 |  |  |
| **474** | SNAI2 |  |  |
| **475** | SNW1 |  |  |
| **476** | SOD1 |  |  |
| **477** | SOD2 |  |  |
| **478** | SORT1 |  |  |
| **479** | SP100 |  |  |
| **480** | SRC |  |  |
| **481** | SRPX |  |  |
| **482** | SST |  |  |
| **483** | SSTR3 |  |  |
| **484** | ST20 |  |  |
| **485** | STK11 |  |  |
| **486** | STK24 |  |  |
| **487** | STK25 |  |  |
| **488** | STK3 |  |  |
| **489** | STK4 |  |  |
| **490** | STRADB |  |  |
| **491** | STX4 |  |  |
| **492** | STYXL1 |  |  |
| **493** | SYVN1 |  |  |
| **494** | TAF9 |  |  |
| **495** | TAF9B |  |  |
| **496** | TCF7L2 |  |  |
| **497** | TERT |  |  |
| **498** | TFDP1 |  |  |
| **499** | TFDP2 |  |  |
| **500** | TFPT |  |  |
| **501** | TGFB1 |  |  |
| **502** | TGFB2 |  |  |
| **503** | TGFBR1 |  |  |
| **504** | THBS1 |  |  |
| **505** | TICAM1 |  |  |
| **506** | TICAM2 |  |  |
| **507** | TIMM50 |  |  |
| **508** | TIMP3 |  |  |
| **509** | TLR3 |  |  |
| **510** | TLR4 |  |  |
| **511** | TM2D1 |  |  |
| **512** | TMBIM1 |  |  |
| **513** | TMBIM6 |  |  |
| **514** | TMC8 |  |  |
| **515** | TMEM102 |  |  |
| **516** | TMEM109 |  |  |
| **517** | TMEM117 |  |  |
| **518** | TMEM14A |  |  |
| **519** | TMEM161A |  |  |
| **520** | ZSWIM2 |  |  |
| **521** | TNFAIP3 |  |  |
| **522** | TNFRSF10A |  |  |
| **523** | TNFRSF10B |  |  |
| **524** | TNFRSF10C |  |  |
| **525** | TNFRSF12A |  |  |
| **526** | TNFRSF1A |  |  |
| **527** | TNFRSF1B |  |  |
| **528** | TNFRSF25 |  |  |
| **529** | TNFSF10 |  |  |
| **530** | TNFSF12 |  |  |
| **531** | TOPORS |  |  |
| **532** | TP53 |  |  |
| **533** | TP53BP2 |  |  |
| **534** | TP63 |  |  |
| **535** | TP73 |  |  |
| **536** | TPD52L1 |  |  |
| **537** | TPT1 |  |  |
| **538** | TRADD |  |  |
| **539** | TRAF1 |  |  |
| **540** | TRAF2 |  |  |
| **541** | TRAF7 |  |  |
| **542** | TRAP1 |  |  |
| **543** | TRIAP1 |  |  |
| **544** | TRIB3 |  |  |
| **545** | TRIM32 |  |  |
| **546** | TRIM39 |  |  |
| **547** | TXNDC12 |  |  |
| **548** | TYROBP |  |  |
| **549** | UACA |  |  |
| **550** | UBB |  |  |
| **551** | UBE2K |  |  |
| **552** | UBE4B |  |  |
| **553** | UBQLN1 |  |  |
| **554** | UMOD |  |  |
| **555** | UNC5B |  |  |
| **556** | URI1 |  |  |
| **557** | USP28 |  |  |
| **558** | USP47 |  |  |
| **559** | VDAC2 |  |  |
| **560** | VNN1 |  |  |
| **561** | WDR35 |  |  |
| **562** | WNT4 |  |  |
| **563** | WWOX |  |  |
| **564** | XBP1 |  |  |
| **565** | YAP1 |  |  |
| **566** | YBX3 |  |  |
| **567** | YWHAB |  |  |
| **568** | YWHAE |  |  |
| **569** | YWHAG |  |  |
| **570** | YWHAH |  |  |
| **571** | YWHAQ |  |  |
| **572** | YWHAZ |  |  |
| **573** | ZC3HC1 |  |  |
| **574** | ZDHHC3 |  |  |
| **575** | ZMYND11 |  |  |
| **576** | ZNF205 |  |  |
| **577** | ZNF385A |  |  |


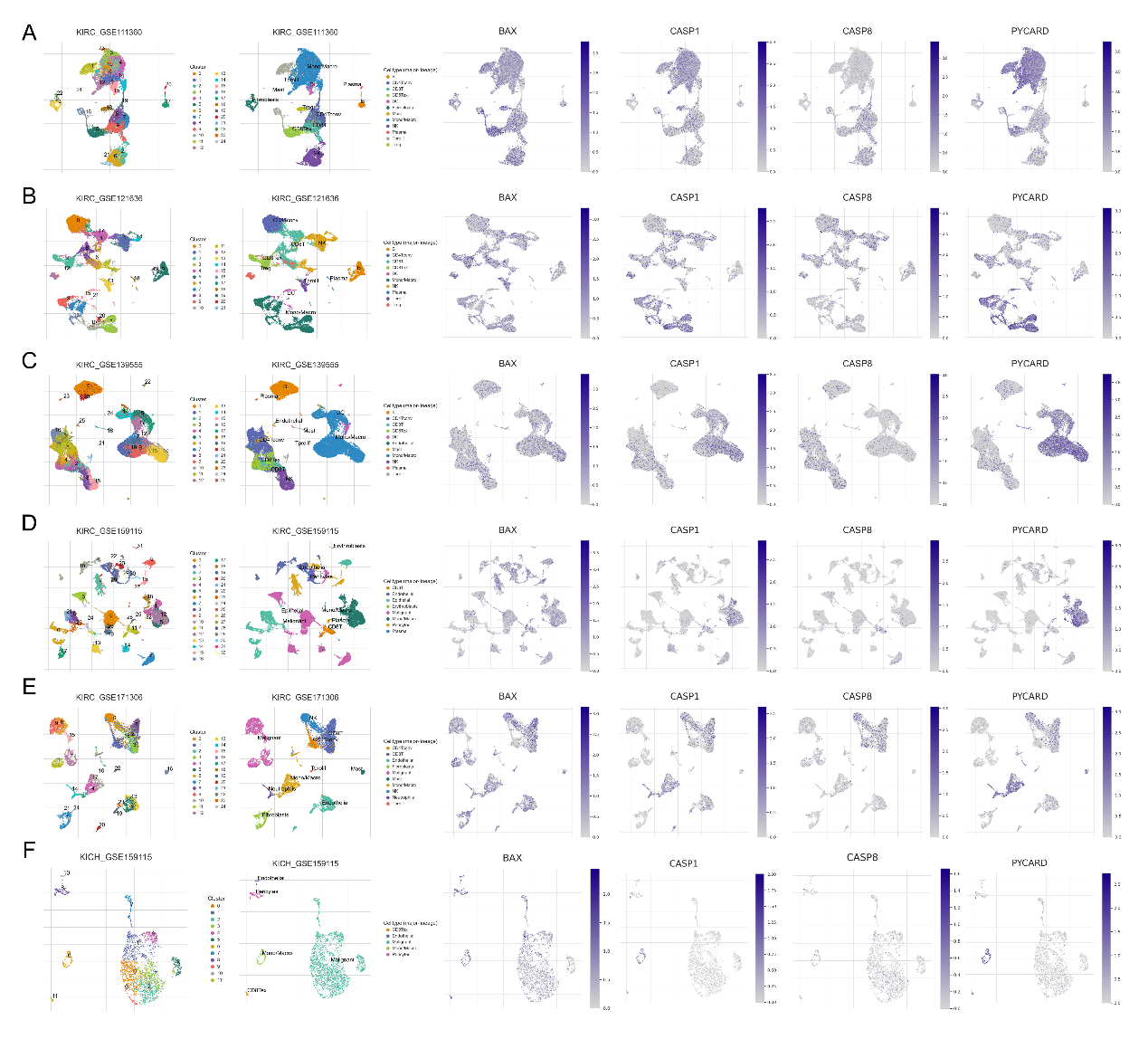


**Figure S1. Single-cell sequencing analysis.** UMAP plots of single-cell sequencing datasets and cellular localization of BAX, CASP1, CASP8, and PYCARD in KIRC (A-E), KICH (F).


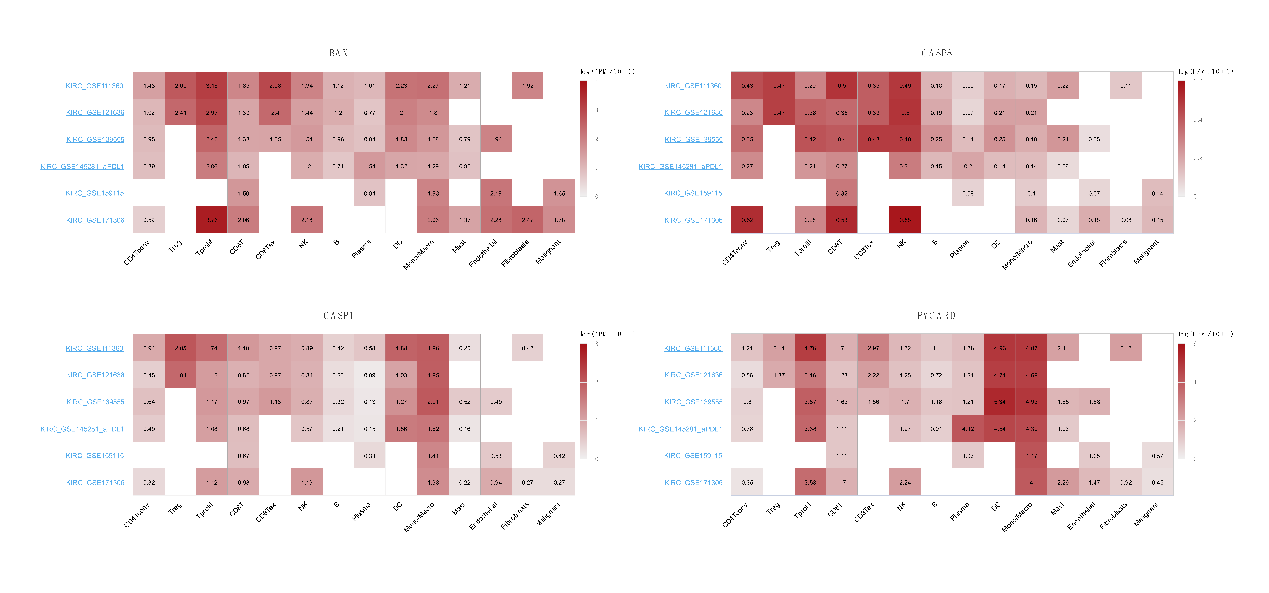


**Figure S2 Heatmap of expression in BAX, CASP1, CASP8 and PYCARD single-cell datasets.**


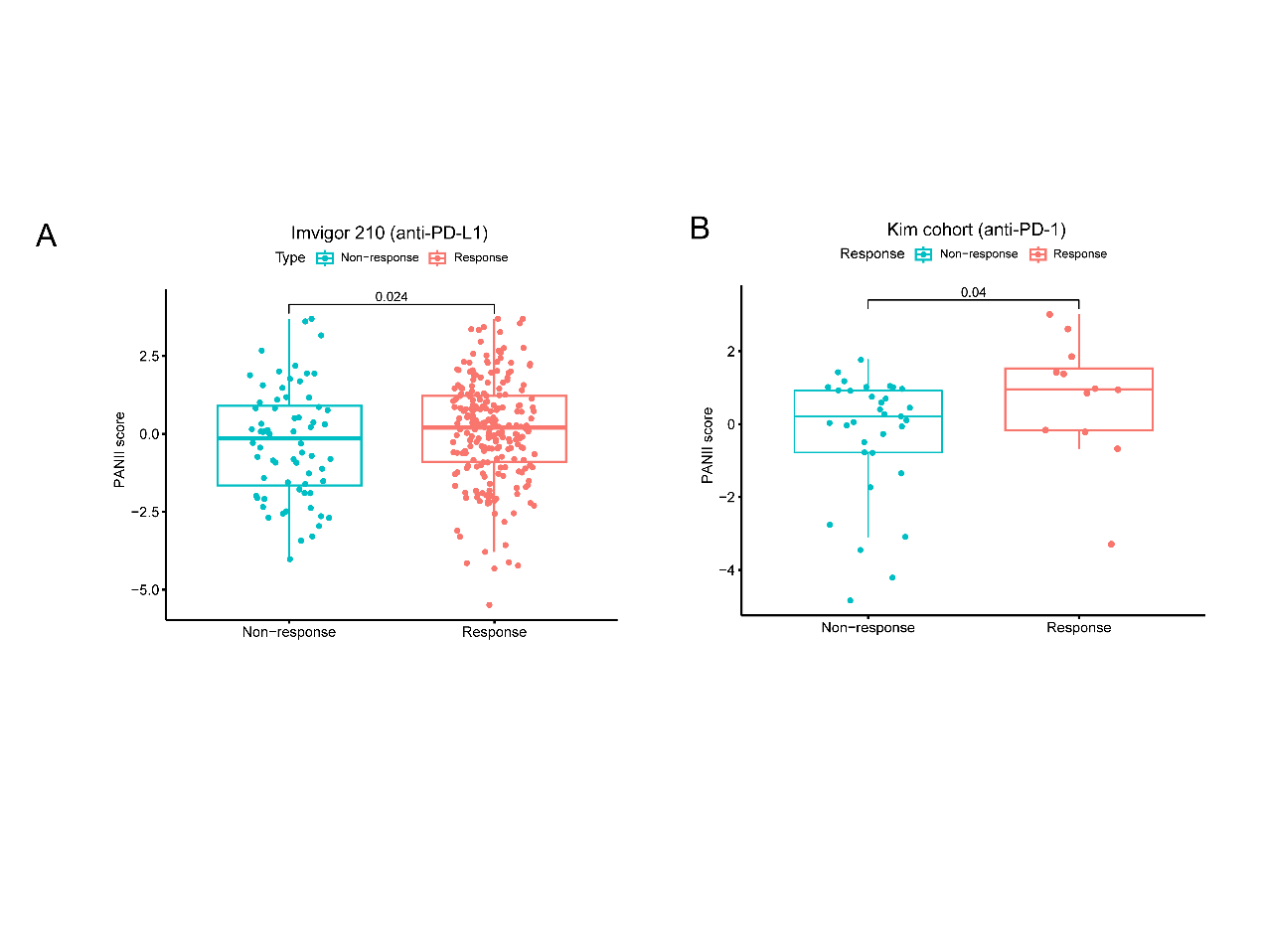


**Figure S3 External immunotherapy dataset validation.**


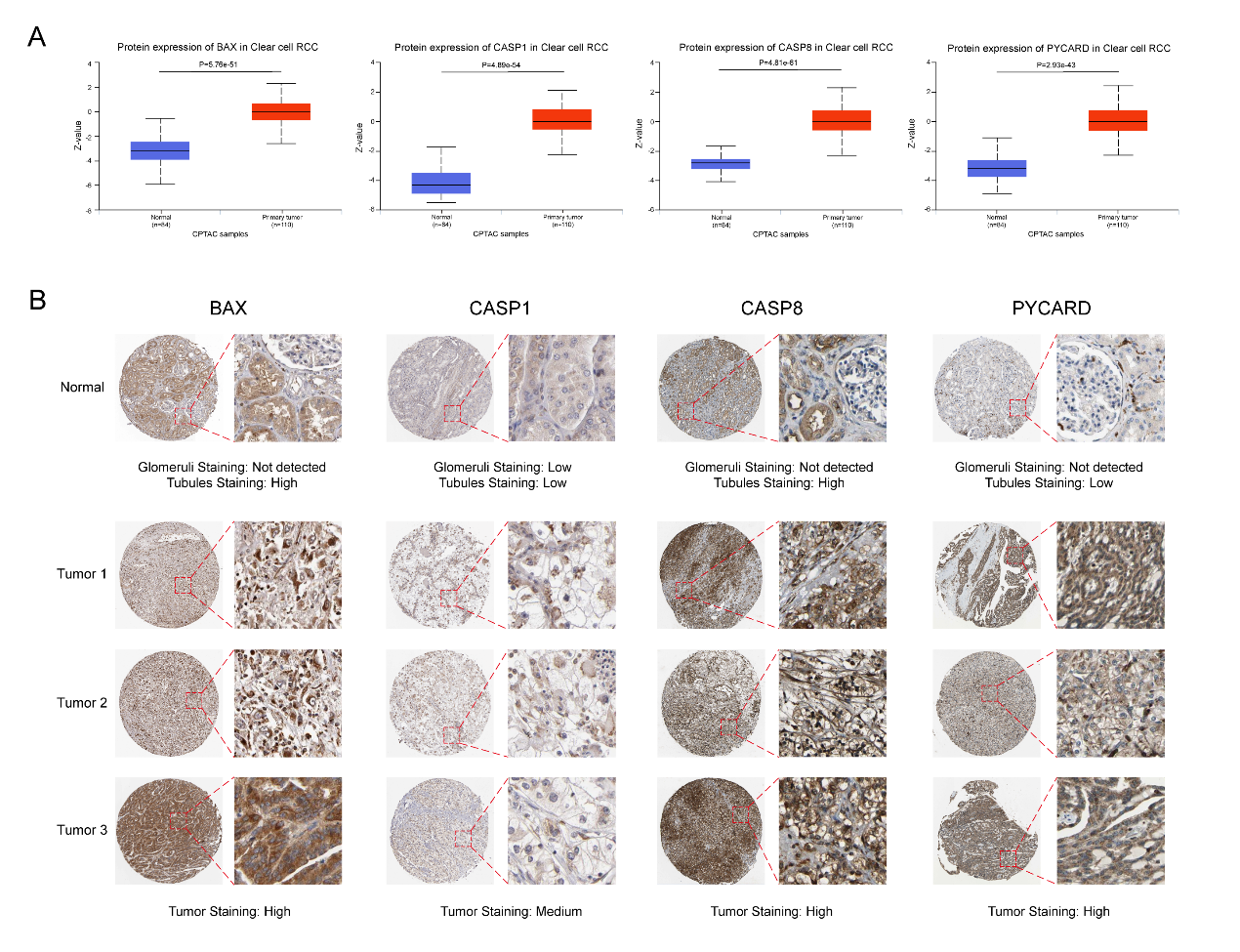


**Figure S4. Protein expression levels and Immunohistochemical.** (A) Differences in protein expression of BAX, CASP1, CASP8 and PYCARD in renal clear cells. (B) Immunohistochemical staining of BAX, CASP1, CASP8 and PYCARD in renal normal tissue and renal clear cell carcinoma.
